# Bis[tris(propane-1,3-diamine-κ^2^ *N*,*N*′)­nickel(II)] di­aqua­bis(propane-1,3-di­amine-κ^2^ *N*,*N*′)nickel(II) hexa­bromide dihydrate

**DOI:** 10.1107/S1600536814011052

**Published:** 2014-05-21

**Authors:** Aymen Yangui, Walid Rekik, Slim Elleuch, Younes Abid

**Affiliations:** a Laboratoire de Physique Appliquée, Faculté des Sciences de Sfax, Université de Sfax, BP 1171, 3018 Sfax, Tunisia; b Laboratoire Physico-Chimie de l’Etat Solide, Département de Chimie, Faculté des Sciences de Sfax, Université de Sfax, BP 1171, 3018 Sfax, Tunisia

## Abstract

In the title compound, [Ni(C_3_H_10_N_2_)_3_]_2_[Ni(C_3_H_10_N_2_)_2_(H_2_O)_2_]Br_6_·2H_2_O, one Ni^2+^ cation, located on an inversion centre, is coordinated by four N atoms from two ligands and by two water O atoms. The other Ni^2+^ cation, located in a general position, is coordinated by six N atoms from three ligands. In both cases, the Ni^2+^ cation has an octa­hedral coordination environment. The overall structural cohesion is ensured by three types of hydrogen bonds, N—H⋯Br, O—H⋯Br and O—H⋯O, which connect the two types of complex cations, the bromide counter-anions and the lattice water molecules into a three-dimensional network.

## Related literature   

For the multiple coordination modes of amine derivatives as ligands to metal ions, see: Manzur *et al.* (2007 ▶); Ismayilov *et al.* (2007 ▶); Austria *et al.* (2007 ▶). For control of the aggregation of mol­ecules or ions in the solid state in crystal engineering, see: Burrows (2004 ▶). For hydrogen bonding in bifunctional ligands, see: Simard *et al.* (1991 ▶); Zerkowski & Whitesides (1994 ▶).
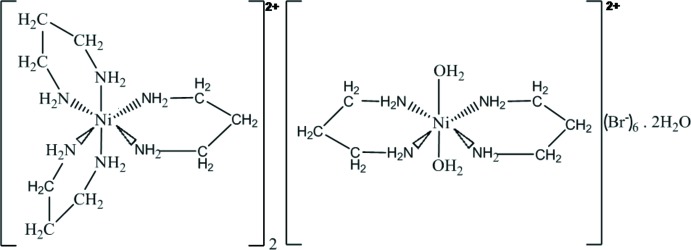


## Experimental   

### 

#### Crystal data  

[Ni(C_3_H_10_N_2_)_3_]_2_[Ni(C_3_H_10_N_2_)_2_(H_2_O)_2_]Br_6_·2H_2_O
*M* *_r_* = 1320.57
Triclinic, 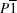

*a* = 8.760 (5) Å
*b* = 13.327 (5) Å
*c* = 13.387 (5) Å
α = 107.774 (5)°
β = 109.045 (5)°
γ = 99.504 (5)°
*V* = 1344.6 (11) Å^3^
*Z* = 1
Mo *K*α radiation
μ = 5.54 mm^−1^
*T* = 296 K
0.36 × 0.30 × 0.16 mm

#### Data collection  

Nonius KappaCCD diffractometer
Absorption correction: analytical (de Meulenaer & Tompa, 1965 ▶) *T*_min_ = 0.215, *T*_max_ = 0.330
17594 measured reflections
8674 independent reflections
5022 reflections with *I* > 2σ(*I*)
*R*_int_ = 0.028

#### Refinement  

*R*[*F* ^2^ > 2σ(*F* ^2^)] = 0.038
*wR*(*F* ^2^) = 0.084
*S* = 1.00
8674 reflections
257 parameters
6 restraints
H atoms treated by a mixture of independent and constrained refinement
Δρ_max_ = 0.90 e Å^−3^
Δρ_min_ = −0.74 e Å^−3^

### 

Data collection: *COLLECT* (Nonius, 1998 ▶); cell refinement: *HKL *SCALEPACK** (Otwinowski & Minor 1997 ▶); data reduction: *HKL *DENZO** (Otwinowski & Minor 1997 ▶) and *HKL *SCALEPACK**; program(s) used to solve structure: *SHELXS2013* (Sheldrick, 2008 ▶); program(s) used to refine structure: *SHELXL2013* (Sheldrick, 2008 ▶); molecular graphics: *DIAMOND* (Brandenburg & Berndt, 1999 ▶); software used to prepare material for publication: *WinGX* (Farrugia, 2012 ▶).

## Supplementary Material

Crystal structure: contains datablock(s) global, I. DOI: 10.1107/S1600536814011052/rk2424sup1.cif

Structure factors: contains datablock(s) I. DOI: 10.1107/S1600536814011052/rk2424Isup2.hkl

CCDC reference: 1002808

Additional supporting information:  crystallographic information; 3D view; checkCIF report

## Figures and Tables

**Table 1:** Hydrogen-bond geometry (Å, °)

*D*—H⋯*A*	*D*—H	H⋯*A*	*D*⋯*A*	*D*—H⋯*A*
O1—H1⋯O2	0.84 (2)	1.98 (2)	2.805 (4)	169 (4)
O1—H2⋯Br3^i^	0.84 (2)	2.37 (2)	3.208 (3)	170 (3)
O2—H4⋯Br2	0.84 (2)	2.55 (2)	3.327 (3)	154 (4)
O2—H3⋯Br1	0.83 (2)	2.61 (2)	3.443 (3)	174 (3)
N1—H1*A*⋯Br2^ii^	0.97	2.58	3.541 (3)	170
N1—H1*B*⋯Br3^i^	0.97	2.90	3.699 (3)	141
N2—H2*D*⋯Br3^iii^	0.97	2.77	3.630 (3)	149
N2—H2*C*⋯Br2	0.97	3.02	3.720 (3)	130
N3—H3*A*⋯Br3	0.97	2.73	3.467 (3)	133
N3—H3*B*⋯Br2^iii^	0.97	2.70	3.544 (3)	146
N4—H4*A*⋯Br1^iv^	0.97	2.70	3.644 (3)	163
N4—H4*B*⋯Br3^v^	0.97	2.72	3.646 (3)	161
N5—H5*A*⋯Br1^vi^	0.97	2.64	3.488 (2)	146
N5—H5*B*⋯Br2^iii^	0.97	2.63	3.537 (3)	156
N6—H6*A*⋯Br1	0.97	2.49	3.445 (3)	170
N6—H6*B*⋯Br1^iv^	0.97	2.55	3.504 (3)	169
N7—H7*B*⋯Br2^iii^	0.97	2.75	3.615 (3)	149
N7—H7*A*⋯Br2	0.97	2.99	3.726 (3)	133
N8—H8*A*⋯Br3^v^	0.97	2.59	3.558 (3)	175
N8—H8*B*⋯Br1	0.97	2.85	3.768 (3)	159

Symmetry codes: (i) 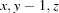; (ii) 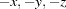; (iii) 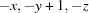; (iv) 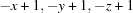; (v) 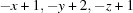; (vi) 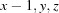.

**Fig. 1.**
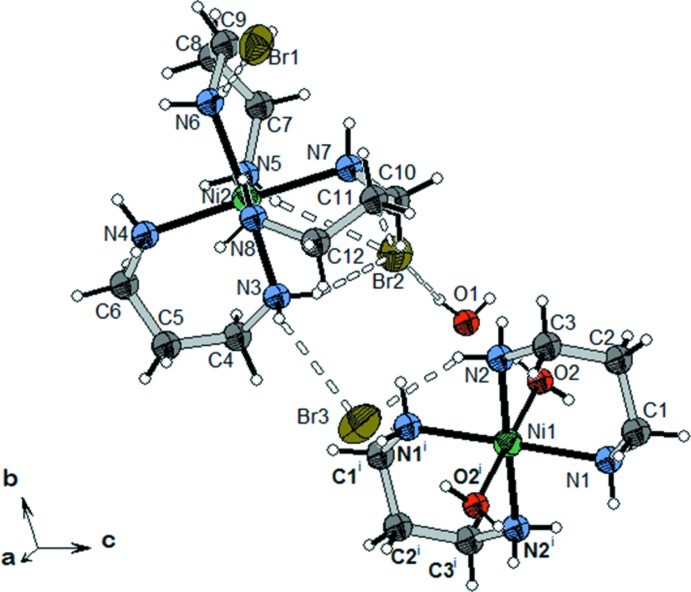
Asymmetric unit of the structure of I extended by symmetry to give complete octahedron environment nickel atom. Displacement ellipsoids are presented at the 50% probability level. H atoms are presented as a small spheres of arbitrary radius. Symmetry code: (i) -x, -y, -z.

**Fig. 2.**
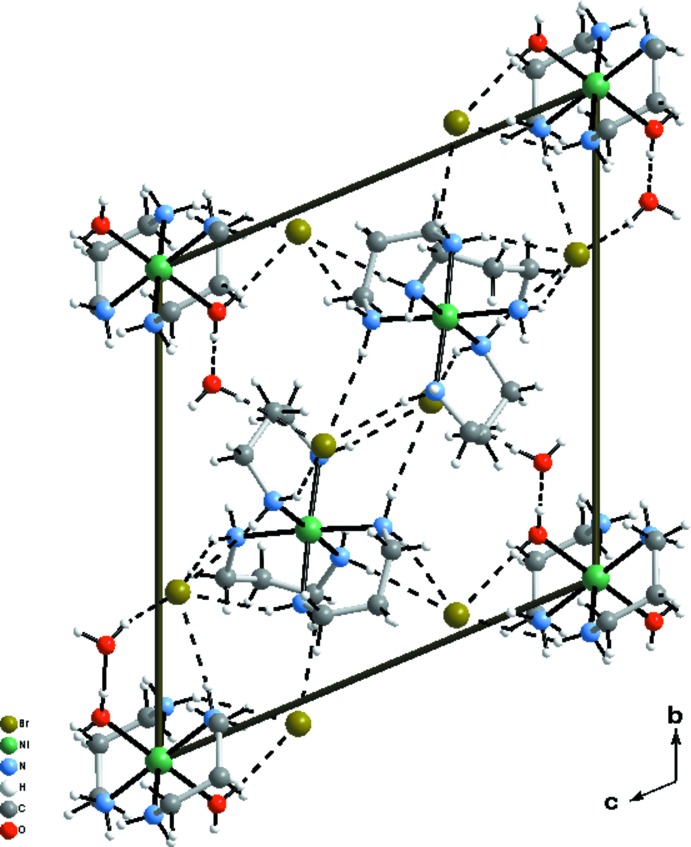
Projection of the structure of I along the a axis.

## supplementary crystallographic information

### 1. Comment

Compounds having specific functional groups have received considerable attention due to their particular properties and applications. For example, derivatives of the amino acids have a biological activity and amine derivatives have potential ability to form metal–organic frameworks because of their multiple coordination modes as ligands to metal ions (Austria *et al.*, 2007; Ismayilov *et al.*, 2007; Manzur *et al.*, 2007). The use of hydrogen bonds to control the aggregation of molecules or ions in the solid state is a key tool in crystal engineering (Burrows, 2004). Although such concepts were originally developed for organic systems, many studies have extended these ideas into the inorganic domain by using bifunctional ligands that are capable of simultaneously coordinating to a metal centre and presenting one or more hydrogen bonding (Simard *et al.*, 1991; Zerkowski *et al.*, 1994). In this context, we report here the chemical preparation and the crystal structure of a novel hybrid material using nickel as transition metal presenting the following formula [Ni(C_3_N_2_H_10_)_2_(H_2_O)_2_][Ni(C_3_N_2_H_10_)_3_]_2_Br_6_·2H_2_O, (I). The asymmetric unit of I, represented in Fig. 1, contains two crystallographically independent nickel atoms. The first one occupies a general position and it is coordinated by three 1,3–diaminopropane molecules amine, which are bidentate ligands. The second type of nickel atom lies in a special position on inversion centre and it is coordinated by one molecule amine and one water molecule and their symmetric by the the inversion centre. Consequently, the nickel atoms, in this compound, adopt two different octahedral coordination. The asymmetric unit of I conatins also two free water molecules and three bromine ions. As it can be seen in Fig. 2, the cohesion of the crystal structure is ensured by three types of hydrogen bonds, N—H···Br, O—H···Br and O—H···O, established between the different entities ginving rise to a three dimensional H–bonds network.

### 2. Experimental

The title compound is resulting from a chemical reaction between three reagents: 1,3–diaminopropane (C_3_H_10_N_2_), hydrobromic acid (HBr) and nickel bromide (NiBr_2_). The 1 mmol of NiBr_2_ and 1 mmol of the diamine with excess of HBr were mixed in the *DMF* solvent. The obtained solution is kept at room temperature. After 4 days, purple platelets were formed. The purity of the product was improved by a second recrystallization.

### 3. Refinement

The water H atoms were located in difference map and refined with O—H distance restraints of 0.85 (2)Å and H···H distance restraints of 1.35 (2)Å. The H atoms bonded to C and N atoms were positioned geometrically (with distances C—H = 0.97Å and N—H = 0.90Å) allowed to ride on their parent atoms, with *U*_iso_ = 1.2*U*_eq_(C, N).

### Crystal data

**Table:**

[Ni(C_3_H_10_N_2_)_3_]_2_[Ni(C_3_H_10_N_2_)_2_(H_2_O)_2_]Br_6_·2H_2_O	*Z* = 1
*M**_r_* = 1320.57	*F*(000) = 670
Triclinic, *P*1	*D*_x_ = 1.631 Mg m^−^^3^
*a* = 8.760 (5) Å	Mo *K*α radiation, λ = 0.71073 Å
*b* = 13.327 (5) Å	Cell parameters from 17594 reflections
*c* = 13.387 (5) Å	θ = 1.7–31.2°
α = 107.774 (5)°	µ = 5.54 mm^−^^1^
β = 109.045 (5)°	*T* = 296 K
γ = 99.504 (5)°	Pellets, purple
*V* = 1344.6 (11) Å^3^	0.36 × 0.30 × 0.16 mm

### Data collection

**Table:**

Nonius KappaCCD diffractometer	8674 independent reflections
Radiation source: fine–focus sealed tube	5022 reflections with *I* > 2σ(*I*)
Horizontally mounted graphite crystal monochromator	*R*_int_ = 0.028
Detector resolution: 9 pixels mm^-1^	θ_max_ = 31.2°, θ_min_ = 1.7°
rotation images, thick slices scans	*h* = −12→12
Absorption correction: analytical (de Meulenaer & Tompa, 1965)	*k* = −19→18
*T*_min_ = 0.215, *T*_max_ = 0.330	*l* = −19→18
17594 measured reflections	

### Refinement

**Table:**

Refinement on *F*^2^	Primary atom site location: structure-invariant direct methods
Least-squares matrix: full	Secondary atom site location: difference Fourier map
*R*[*F*^2^ > 2σ(*F*^2^)] = 0.038	Hydrogen site location: inferred from neighbouring sites
*wR*(*F*^2^) = 0.084	H atoms treated by a mixture of independent and constrained refinement
*S* = 1.00	*w* = 1/[σ^2^(*F*_o_^2^) + (0.0337*P*)^2^ + 0.1194*P*] where *P* = (*F*_o_^2^ + 2*F*_c_^2^)/3
8674 reflections	(Δ/σ)_max_ = 0.001
257 parameters	Δρ_max_ = 0.90 e Å^−^^3^
6 restraints	Δρ_min_ = −0.74 e Å^−^^3^

### Special details

**Table:**

Geometry. All s.u.'s (except the s.u. in the dihedral angle between two l.s. planes) are estimated using the full covariance matrix. The cell s.u.'s are taken into account individually in the estimation of s.u.'s in distances, angles and torsion angles; correlations between s.u.'s in cell parameters are only used when they are defined by crystal symmetry. An approximate (isotropic) treatment of cell s.u.'s is used for estimating s.u.'s involving l.s. planes.
Refinement. Refinement of *F*^2^ against ALL reflections. The weighted *R*–factor *wR* and goodness of fit *S* are based on *F*^2^, conventional *R*–factors *R* are based on *F*, with *F* set to zero for negative *F*^2^. The threshold expression of *F*^2^ > σ(*F*^2^) is used only for calculating *R*–factors(gt) *etc*. and is not relevant to the choice of reflections for refinement. *R*–factors based on *F*^2^ are statistically about twice as large as those based on *F*, and *R*–factors based on ALL data will be even larger.

### Fractional atomic coordinates and isotropic or equivalent isotropic displacement parameters (Å^2^)

**Table:**

	*x*	*y*	*z*	*U*_iso_*/*U*_eq_	
Br1	0.69831 (4)	0.50049 (3)	0.37689 (3)	0.05784 (10)	
Br3	0.38496 (5)	1.04327 (3)	0.31879 (3)	0.07285 (12)	
Ni1	0.0000	0.0000	0.0000	0.03393 (11)	
Ni2	0.24711 (4)	0.65812 (2)	0.34338 (3)	0.03374 (9)	
N3	0.1826 (3)	0.80148 (18)	0.3275 (2)	0.0479 (6)	
H3A	0.2872	0.8609	0.3623	0.057*	
H3B	0.1356	0.7875	0.2464	0.057*	
N4	0.2454 (3)	0.71517 (19)	0.51262 (19)	0.0465 (6)	
H4A	0.2339	0.6523	0.5346	0.056*	
H4B	0.3555	0.7668	0.5650	0.056*	
N5	−0.0146 (3)	0.56993 (17)	0.26203 (19)	0.0424 (5)	
H5A	−0.0588	0.5801	0.3209	0.051*	
H5B	−0.0703	0.6044	0.2122	0.051*	
N6	0.3057 (3)	0.51614 (18)	0.3677 (2)	0.0491 (6)	
H6A	0.4190	0.5213	0.3709	0.059*	
H6B	0.3108	0.5223	0.4429	0.059*	
N7	0.2549 (3)	0.6107 (2)	0.1765 (2)	0.0487 (6)	
H7A	0.2807	0.5409	0.1602	0.058*	
H7B	0.1415	0.5965	0.1215	0.058*	
N8	0.5159 (3)	0.7397 (2)	0.4153 (2)	0.0527 (6)	
H8A	0.5440	0.7955	0.4901	0.063*	
H8B	0.5733	0.6855	0.4278	0.063*	
C9	0.1995 (5)	0.4044 (2)	0.2877 (3)	0.0694 (10)	
H9A	0.2356	0.3520	0.3198	0.083*	
H9B	0.2153	0.3886	0.2167	0.083*	
O2	0.4632 (4)	0.2799 (2)	0.1212 (3)	0.0724 (7)	
C7	−0.0660 (4)	0.4506 (3)	0.1937 (3)	0.0608 (9)	
H7C	−0.0349	0.4388	0.1287	0.073*	
H7D	−0.1878	0.4215	0.1644	0.073*	
C8	0.0148 (5)	0.3893 (3)	0.2623 (3)	0.0762 (11)	
H8C	−0.0453	0.3114	0.2213	0.091*	
H8D	0.0017	0.4128	0.3343	0.091*	
C12	0.3715 (4)	0.6846 (3)	0.1526 (3)	0.0668 (10)	
H12A	0.3364	0.7505	0.1559	0.080*	
H12B	0.3629	0.6478	0.0754	0.080*	
C10	0.5908 (4)	0.7931 (3)	0.3539 (3)	0.0670 (10)	
H10A	0.7124	0.8206	0.3962	0.080*	
H10B	0.5493	0.8558	0.3514	0.080*	
C11	0.5517 (4)	0.7177 (3)	0.2342 (3)	0.0700 (10)	
H11A	0.6220	0.7537	0.2047	0.084*	
H11B	0.5825	0.6515	0.2365	0.084*	
C6	0.1161 (4)	0.7689 (3)	0.5323 (3)	0.0581 (8)	
H6C	0.1345	0.7911	0.6124	0.070*	
H6D	0.0048	0.7160	0.4872	0.070*	
C5	0.1219 (4)	0.8689 (2)	0.5008 (3)	0.0602 (9)	
H5C	0.0513	0.9085	0.5289	0.072*	
H5D	0.2369	0.9174	0.5397	0.072*	
C4	0.0649 (4)	0.8433 (3)	0.3750 (3)	0.0561 (8)	
H4C	−0.0453	0.7888	0.3348	0.067*	
H4D	0.0530	0.9097	0.3614	0.067*	
O1	0.2016 (3)	0.13735 (17)	0.13311 (19)	0.0517 (5)	
N1	−0.0659 (3)	−0.03912 (19)	0.1248 (2)	0.0458 (6)	
H1A	−0.0704	−0.1156	0.1105	0.055*	
H1B	0.0263	0.0048	0.1986	0.055*	
C2	−0.2457 (4)	0.0852 (2)	0.1389 (3)	0.0557 (8)	
H2A	−0.1409	0.1416	0.1933	0.067*	
H2B	−0.3326	0.0984	0.1668	0.067*	
C1	−0.2248 (4)	−0.0249 (2)	0.1366 (3)	0.0566 (8)	
H1C	−0.2239	−0.0320	0.2068	0.068*	
H1D	−0.3203	−0.0828	0.0731	0.068*	
C3	−0.2917 (4)	0.0979 (3)	0.0259 (3)	0.0570 (8)	
H3C	−0.3864	0.0353	−0.0318	0.068*	
H3D	−0.3278	0.1637	0.0321	0.068*	
N2	−0.1514 (3)	0.10575 (18)	−0.0122 (2)	0.0446 (6)	
H2C	−0.0768	0.1806	0.0304	0.053*	
H2D	−0.1994	0.0965	−0.0919	0.053*	
Br2	0.12791 (4)	0.32662 (3)	−0.04291 (3)	0.05752 (10)	
H2	0.261 (4)	0.119 (3)	0.184 (2)	0.070 (12)*	
H1	0.270 (4)	0.185 (3)	0.128 (3)	0.108 (17)*	
H3	0.521 (4)	0.330 (2)	0.1855 (18)	0.084 (14)*	
H4	0.405 (5)	0.307 (3)	0.079 (3)	0.111 (19)*	

### Atomic displacement parameters (Å^2^)

**Table:**

	*U*^11^	*U*^22^	*U*^33^	*U*^12^	*U*^13^	*U*^23^
Br1	0.0568 (2)	0.0720 (2)	0.0668 (2)	0.02667 (17)	0.03530 (17)	0.04018 (18)
Br3	0.0733 (2)	0.04978 (19)	0.0567 (2)	−0.00541 (16)	−0.01077 (18)	0.02214 (16)
Ni1	0.0335 (2)	0.0323 (2)	0.0336 (3)	0.00772 (19)	0.0118 (2)	0.01223 (19)
Ni2	0.03470 (18)	0.03048 (17)	0.03582 (19)	0.00762 (13)	0.01576 (15)	0.01176 (14)
N3	0.0499 (14)	0.0408 (13)	0.0612 (16)	0.0171 (11)	0.0269 (13)	0.0235 (12)
N4	0.0570 (15)	0.0414 (13)	0.0385 (13)	0.0122 (11)	0.0188 (12)	0.0137 (10)
N5	0.0420 (13)	0.0409 (12)	0.0389 (13)	0.0028 (10)	0.0187 (11)	0.0106 (10)
N6	0.0630 (16)	0.0460 (14)	0.0553 (16)	0.0250 (12)	0.0345 (14)	0.0252 (12)
N7	0.0516 (15)	0.0519 (14)	0.0452 (14)	0.0109 (12)	0.0265 (12)	0.0169 (12)
N8	0.0403 (14)	0.0534 (15)	0.0570 (16)	0.0084 (12)	0.0165 (12)	0.0178 (12)
C9	0.108 (3)	0.0420 (18)	0.074 (2)	0.0338 (19)	0.049 (2)	0.0228 (17)
O2	0.0647 (17)	0.0603 (16)	0.081 (2)	0.0147 (14)	0.0234 (15)	0.0215 (16)
C7	0.059 (2)	0.0519 (18)	0.051 (2)	−0.0073 (16)	0.0266 (17)	0.0003 (15)
C8	0.099 (3)	0.0324 (16)	0.087 (3)	−0.0027 (18)	0.047 (2)	0.0101 (17)
C12	0.063 (2)	0.089 (3)	0.071 (2)	0.022 (2)	0.039 (2)	0.048 (2)
C10	0.0399 (17)	0.068 (2)	0.095 (3)	0.0065 (16)	0.0271 (19)	0.039 (2)
C11	0.059 (2)	0.093 (3)	0.091 (3)	0.028 (2)	0.049 (2)	0.055 (2)
C6	0.064 (2)	0.064 (2)	0.0399 (17)	0.0155 (17)	0.0264 (16)	0.0068 (15)
C5	0.060 (2)	0.0494 (18)	0.057 (2)	0.0242 (16)	0.0199 (17)	0.0008 (15)
C4	0.0552 (19)	0.0573 (19)	0.066 (2)	0.0295 (16)	0.0289 (17)	0.0248 (16)
O1	0.0493 (13)	0.0416 (12)	0.0456 (13)	0.0006 (10)	0.0058 (11)	0.0136 (10)
N1	0.0493 (14)	0.0446 (13)	0.0431 (14)	0.0090 (11)	0.0194 (12)	0.0181 (11)
C2	0.0550 (19)	0.0469 (17)	0.063 (2)	0.0095 (15)	0.0342 (17)	0.0097 (15)
C1	0.059 (2)	0.0500 (18)	0.061 (2)	0.0045 (15)	0.0353 (17)	0.0156 (15)
C3	0.0442 (17)	0.0560 (19)	0.068 (2)	0.0177 (15)	0.0221 (16)	0.0183 (16)
N2	0.0412 (13)	0.0419 (13)	0.0522 (15)	0.0136 (11)	0.0179 (12)	0.0204 (11)
Br2	0.0688 (2)	0.05470 (19)	0.04544 (18)	0.01940 (16)	0.01714 (16)	0.02018 (14)

### Geometric parameters (Å, º)

**Table:**

Ni1—N2^i^	2.095 (2)	C7—H7C	0.9700
Ni1—N2	2.095 (2)	C7—H7D	0.9700
Ni1—N1^i^	2.112 (2)	C8—H8C	0.9700
Ni1—N1	2.112 (2)	C8—H8D	0.9700
Ni1—O1^i^	2.129 (2)	C12—C11	1.493 (5)
Ni1—O1	2.129 (2)	C12—H12A	0.9700
Ni2—N5	2.127 (2)	C12—H12B	0.9700
Ni2—N6	2.130 (2)	C10—C11	1.497 (5)
Ni2—N3	2.131 (2)	C10—H10A	0.9700
Ni2—N7	2.155 (2)	C10—H10B	0.9700
Ni2—N4	2.165 (2)	C11—H11A	0.9700
Ni2—N8	2.166 (3)	C11—H11B	0.9700
N3—C4	1.476 (4)	C6—C5	1.513 (4)
N3—H3A	0.9700	C6—H6C	0.9700
N3—H3B	0.9700	C6—H6D	0.9700
N4—C6	1.487 (4)	C5—C4	1.499 (4)
N4—H4A	0.9700	C5—H5C	0.9700
N4—H4B	0.9700	C5—H5D	0.9700
N5—C7	1.475 (4)	C4—H4C	0.9700
N5—H5A	0.9700	C4—H4D	0.9700
N5—H5B	0.9700	O1—H2	0.843 (17)
N6—C9	1.465 (4)	O1—H1	0.839 (18)
N6—H6A	0.9700	N1—C1	1.486 (4)
N6—H6B	0.9700	N1—H1A	0.9700
N7—C12	1.481 (4)	N1—H1B	0.9700
N7—H7A	0.9700	C2—C1	1.501 (4)
N7—H7B	0.9700	C2—C3	1.504 (5)
N8—C10	1.472 (4)	C2—H2A	0.9700
N8—H8A	0.9700	C2—H2B	0.9700
N8—H8B	0.9700	C1—H1C	0.9700
C9—C8	1.505 (5)	C1—H1D	0.9700
C9—H9A	0.9700	C3—N2	1.476 (4)
C9—H9B	0.9700	C3—H3C	0.9700
O2—H3	0.834 (17)	C3—H3D	0.9700
O2—H4	0.841 (17)	N2—H2C	0.9700
C7—C8	1.495 (5)	N2—H2D	0.9700
			
N2^i^—Ni1—N2	180.0	C8—C7—H7D	109.2
N2^i^—Ni1—N1^i^	93.54 (9)	H7C—C7—H7D	107.9
N2—Ni1—N1^i^	86.46 (9)	C7—C8—C9	115.0 (3)
N2^i^—Ni1—N1	86.46 (9)	C7—C8—H8C	108.5
N2—Ni1—N1	93.54 (9)	C9—C8—H8C	108.5
N1^i^—Ni1—N1	180.0	C7—C8—H8D	108.5
N2^i^—Ni1—O1^i^	88.38 (10)	C9—C8—H8D	108.5
N2—Ni1—O1^i^	91.62 (10)	H8C—C8—H8D	107.5
N1^i^—Ni1—O1^i^	89.33 (10)	N7—C12—C11	113.5 (3)
N1—Ni1—O1^i^	90.67 (10)	N7—C12—H12A	108.9
N2^i^—Ni1—O1	91.62 (10)	C11—C12—H12A	108.9
N2—Ni1—O1	88.38 (10)	N7—C12—H12B	108.9
N1^i^—Ni1—O1	90.67 (10)	C11—C12—H12B	108.9
N1—Ni1—O1	89.33 (10)	H12A—C12—H12B	107.7
O1^i^—Ni1—O1	180.0	N8—C10—C11	113.4 (3)
N5—Ni2—N6	90.12 (10)	N8—C10—H10A	108.9
N5—Ni2—N3	88.80 (10)	C11—C10—H10A	108.9
N6—Ni2—N3	176.31 (9)	N8—C10—H10B	108.9
N5—Ni2—N7	88.31 (9)	C11—C10—H10B	108.9
N6—Ni2—N7	93.53 (9)	H10A—C10—H10B	107.7
N3—Ni2—N7	89.98 (10)	C12—C11—C10	115.1 (3)
N5—Ni2—N4	93.56 (9)	C12—C11—H11A	108.5
N6—Ni2—N4	89.02 (9)	C10—C11—H11A	108.5
N3—Ni2—N4	87.51 (9)	C12—C11—H11B	108.5
N7—Ni2—N4	176.84 (9)	C10—C11—H11B	108.5
N5—Ni2—N8	175.67 (9)	H11A—C11—H11B	107.5
N6—Ni2—N8	88.21 (10)	N4—C6—C5	112.3 (3)
N3—Ni2—N8	93.10 (10)	N4—C6—H6C	109.1
N7—Ni2—N8	87.80 (10)	C5—C6—H6C	109.1
N4—Ni2—N8	90.41 (10)	N4—C6—H6D	109.1
C4—N3—Ni2	120.60 (19)	C5—C6—H6D	109.1
C4—N3—H3A	107.2	H6C—C6—H6D	107.9
Ni2—N3—H3A	107.2	C4—C5—C6	114.6 (2)
C4—N3—H3B	107.2	C4—C5—H5C	108.6
Ni2—N3—H3B	107.2	C6—C5—H5C	108.6
H3A—N3—H3B	106.8	C4—C5—H5D	108.6
C6—N4—Ni2	119.51 (19)	C6—C5—H5D	108.6
C6—N4—H4A	107.4	H5C—C5—H5D	107.6
Ni2—N4—H4A	107.4	N3—C4—C5	113.1 (3)
C6—N4—H4B	107.4	N3—C4—H4C	109.0
Ni2—N4—H4B	107.4	C5—C4—H4C	109.0
H4A—N4—H4B	107.0	N3—C4—H4D	109.0
C7—N5—Ni2	118.69 (19)	C5—C4—H4D	109.0
C7—N5—H5A	107.6	H4C—C4—H4D	107.8
Ni2—N5—H5A	107.6	Ni1—O1—H2	112 (2)
C7—N5—H5B	107.6	Ni1—O1—H1	129 (3)
Ni2—N5—H5B	107.6	H2—O1—H1	105 (2)
H5A—N5—H5B	107.1	C1—N1—Ni1	120.33 (19)
C9—N6—Ni2	121.6 (2)	C1—N1—H1A	107.2
C9—N6—H6A	106.9	Ni1—N1—H1A	107.2
Ni2—N6—H6A	106.9	C1—N1—H1B	107.2
C9—N6—H6B	106.9	Ni1—N1—H1B	107.2
Ni2—N6—H6B	106.9	H1A—N1—H1B	106.9
H6A—N6—H6B	106.7	C1—C2—C3	115.4 (3)
C12—N7—Ni2	120.5 (2)	C1—C2—H2A	108.4
C12—N7—H7A	107.2	C3—C2—H2A	108.4
Ni2—N7—H7A	107.2	C1—C2—H2B	108.4
C12—N7—H7B	107.2	C3—C2—H2B	108.4
Ni2—N7—H7B	107.2	H2A—C2—H2B	107.5
H7A—N7—H7B	106.8	N1—C1—C2	111.9 (2)
C10—N8—Ni2	120.6 (2)	N1—C1—H1C	109.2
C10—N8—H8A	107.2	C2—C1—H1C	109.2
Ni2—N8—H8A	107.2	N1—C1—H1D	109.2
C10—N8—H8B	107.2	C2—C1—H1D	109.2
Ni2—N8—H8B	107.2	H1C—C1—H1D	107.9
H8A—N8—H8B	106.8	N2—C3—C2	113.4 (3)
N6—C9—C8	112.7 (3)	N2—C3—H3C	108.9
N6—C9—H9A	109.1	C2—C3—H3C	108.9
C8—C9—H9A	109.1	N2—C3—H3D	108.9
N6—C9—H9B	109.1	C2—C3—H3D	108.9
C8—C9—H9B	109.1	H3C—C3—H3D	107.7
H9A—C9—H9B	107.8	C3—N2—Ni1	120.99 (18)
H3—O2—H4	109 (3)	C3—N2—H2C	107.1
N5—C7—C8	112.1 (3)	Ni1—N2—H2C	107.1
N5—C7—H7C	109.2	C3—N2—H2D	107.1
C8—C7—H7C	109.2	Ni1—N2—H2D	107.1
N5—C7—H7D	109.2	H2C—N2—H2D	106.8

Symmetry code: (i) −*x*, −*y*, −*z*.

### Hydrogen-bond geometry (Å, º)

**Table:**

*D*—H···*A*	*D*—H	H···*A*	*D*···*A*	*D*—H···*A*
O1—H1···O2	0.84 (2)	1.98 (2)	2.805 (4)	169 (4)
O1—H2···Br3^ii^	0.84 (2)	2.37 (2)	3.208 (3)	170 (3)
O2—H4···Br2	0.84 (2)	2.55 (2)	3.327 (3)	154 (4)
O2—H3···Br1	0.83 (2)	2.61 (2)	3.443 (3)	174 (3)
N1—H1*A*···Br2^i^	0.97	2.58	3.541 (3)	170
N1—H1*B*···Br3^ii^	0.97	2.90	3.699 (3)	141
N2—H2*D*···Br3^iii^	0.97	2.77	3.630 (3)	149
N2—H2*C*···Br2	0.97	3.02	3.720 (3)	130
N3—H3*A*···Br3	0.97	2.73	3.467 (3)	133
N3—H3*B*···Br2^iii^	0.97	2.70	3.544 (3)	146
N4—H4*A*···Br1^iv^	0.97	2.70	3.644 (3)	163
N4—H4*B*···Br3^v^	0.97	2.72	3.646 (3)	161
N5—H5*A*···Br1^vi^	0.97	2.64	3.488 (2)	146
N5—H5*B*···Br2^iii^	0.97	2.63	3.537 (3)	156
N6—H6*A*···Br1	0.97	2.49	3.445 (3)	170
N6—H6*B*···Br1^iv^	0.97	2.55	3.504 (3)	169
N7—H7*B*···Br2^iii^	0.97	2.75	3.615 (3)	149
N7—H7*A*···Br2	0.97	2.99	3.726 (3)	133
N8—H8*A*···Br3^v^	0.97	2.59	3.558 (3)	175
N8—H8*B*···Br1	0.97	2.85	3.768 (3)	159

Symmetry codes: (i) −*x*, −*y*, −*z*; (ii) *x*, *y*−1, *z*; (iii) −*x*, −*y*+1, −*z*; (iv) −*x*+1, −*y*+1, −*z*+1; (v) −*x*+1, −*y*+2, −*z*+1; (vi) *x*−1, *y*, *z*.
